# Owl Pellet Content Analysis Proves an Effective Technique to Monitor a Population of Threatened Julia Creek Dunnarts (*Sminthopsis douglasi*) Throughout a Native Rodent Plague

**DOI:** 10.1002/ece3.70922

**Published:** 2025-02-20

**Authors:** Cameron L. Charley, Emma L. Gray, Andrew M. Baker

**Affiliations:** ^1^ School of Biology and Environmental Science Queensland University of Technology Brisbane Queensland Australia; ^2^ Biodiversity and Geosciences Program, Queensland Museum South Brisbane Queensland Australia

**Keywords:** Dasyuridae, detection, dietary analysis, non‐invasive survey method, small mammal, temporal dynamics

## Abstract

Logistical, environmental and temporal considerations can limit the effectiveness of long‐term live trapping for small mammals in remote environments. Owl pellet content analysis offers a low‐cost, non‐invasive alternative to live trapping, as it is generally reflective of prey abundance within the broader small mammal community. One species to which this detection technique could be readily applied is the threatened Australian dasyurid, the Julia Creek dunnart, *Sminthopsis douglasi* . Most population information is outdated, and the species is notoriously difficult to monitor. Here, we aimed to monitor *S. douglasi* and other small terrestrial vertebrates over time and in relation to a native long‐haired rat ( *Rattus villosissimus* ) plague, assessing their occurrence as dietary items in eastern barn owl ( *Tyto javanica delicatula* ) pellets collected at Toorak, north‐west Queensland, Australia. A total of 1007 individual vertebrates were identified from 706 barn owl pellets spanning 3 present‐day collections (2023–2024), with further analysis incorporating a prior published historical dataset (1994–2001, 210 pellets). We demonstrated a shift in Toorak small mammal community structure both over time and in response to an active *R. villosissimus* plague. Despite declines across present‐day pellet collections, *S. douglasi* was always detected in high abundance, peaking at 30.75% of all individuals. Cumulative probability of detection indicated that analysis of owl pellets was highly effective at detecting *S. douglasi* (within 20 pellets) despite the ongoing rodent plague, which has undermined the effectiveness of parallel live trapping efforts across the region. Owl pellet analysis is thus an effective methodology for rapidly assessing *S. douglasi* populations and should be incorporated into both *S. douglasi* and other small mammal species monitoring regimes.

## Graphical abstract

Julia Creek dunnart ( *Sminthopsis douglasi* ) craniodental material from loose eastern barn owl ( *Tyto javanica delicatula* ) pellets collected from Toorak, Queensland, Australia. Photo: Cameron L. Charley.

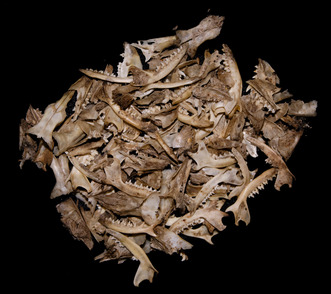

## 1 Introduction

The evidence is now overwhelming that Earth is in the midst of a human‐driven, sixth mass extinction, which has led to major ongoing losses in worldwide biodiversity (Ceballos, Ehrlich, and Raven 2020; Cowie, Bouchet, and Fontaine 2022; McCallum 2015). Of particular concern are terrestrial small mammal fauna, for which European colonisation in places such as Australia has triggered accelerated rates of extinction, primarily driven by introduced predators, changed fire regimes and habitat modification (Bilney, Cooke, and White 2010; Johnson and Isaac 2009; Woinarski, Burbidge, and Harrison 2015). Preventing further extinctions is of utmost importance to biodiversity conservation, and monitoring is critical to the success of such efforts (Tilman et al. 2017; Woinarski, Garnett, and Legge in press). Monitoring provides the baseline measurement of populations and their trends (Kearney, Kern, and Kutt 2020; Kutt, Kearney, and Kern 2021; Scheele et al. 2019) and acts as a foundation to evaluate the effectiveness of conservation implementation (Butchart et al. 2010; Garnett et al. 2019; Lavery et al. 2021). In short, inadequate monitoring compromises conservation (Burns et al. 2014; Scheele et al. 2019), and unfortunately, Australia has a poor track record of successful biodiversity monitoring, with many threatened species often either without an active monitoring program or monitored poorly (Lindenmayer and Gibbons 2012; Preece and Fitzsimons 2022; Scheele et al. 2019). As a result, a limited understanding of population ecology for many Australian threatened species is a key limiting factor in their conservation (Burns et al. 2014; Eyre et al. 2011; Lavery et al. 2021; Tomlinson et al. 2023).

In Australia, live capture metal box (Elliott‐style) traps are the gold standard of small mammal monitoring (Australian Government 2011; Tasker and Dickman 2001). However, despite being highly successful and widely adopted, live trapping can be time consuming, inefficient and expensive (Freeman et al. 2022; Harkins, Keinath, and Ben‐David 2019; Peralta et al. 2023; Perkins et al. 2013; Thompson and Thompson 2007). Indeed, for targeting some species, including dunnarts, live trapping alone may be largely unsuccessful (Catling, Burt, and Kooyman 1997; Mifsud 1999; Murphy 2016; Thomas et al. 2020). It is also confined to small spatial and temporal scales, which are generally more vulnerable to logistical constraints (Bakker, Patterson, et al. 2024; Bakker, Schoenefuss, et al. 2024; Letnic and Dickman 2006; Pavey, Nano, and Waltert 2020; Perkins et al. 2013).

The Australian continent is large, climatically variable and spatially complex (Smyth and James 2004). Rainfall driven population dynamics of fauna are common throughout arid and semi‐arid ecosystems, which can lead to drastic shifts in small mammal community structure over time (Letnic and Dickman 2010; Morton et al. 2011), particularly in relation to irruptive rodent species (Greenville, Wardle, and Dickman 2012; Thibault et al. 2010). Most long‐term monitoring regimes that utilise trapping in such regions typically only monitor a few times a year for a limited number of days at a time (e.g., Dickman et al. 2001; Greenville et al. 2016; Pavey and Nano 2013; Pavey, Nano, and Waltert 2020), although the ecological processes of target species generally operate over much longer periods (Kelly et al. 2013). Additionally, fieldwork is usually confined to favourable seasons (Bakker, Patterson, et al. 2024). As a result, such studies may inadequately represent temporal variation in small mammal communities, particularly given relationships may already be unclear, such as in relation to weather events, seasonal patterns and boom‐bust cycles (Pavey, Nano, and Waltert 2020; Schoenefuss et al. 2024). Therefore, ideal detection methodologies would cover longer spatiotemporal scales, while at the same time remaining simple enough to be cost‐effective and easily measured (Smyth and James 2004; Watson and Novelly 2004).

Low‐cost, non‐invasive detection techniques are useful alternatives to traditional trapping for small mammal species (Thomas et al. 2020). One such method is owl pellet content analysis (henceforth, owl pellets), which in Australia often utilises the eastern barn owl ( *Tyto javanica delicatula* ) (e.g., Debus, Olsen, and Rose 2004; Heywood and Pavey 2002; Schoenefuss et al. 2024), although other raptor species may be employed (e.g., Clulow et al. 2011; Debus, Ley, and Rose 2007; Jiménez‐Nájar et al. 2021; Viteri, Stegner, and Hadly 2022). This technique focuses on the morphological identification of bones found in the regurgitated prey remains of owls, deposited at the roost as pellets. The idiosyncratic morphological traits of mammal craniodental material (Archer 1981) readily enables identification of most individuals to species (Kutt et al. 2021; Schoenefuss et al. 2024). Barn owls are assumed to utilise home ranges of 1000 ha or more (Kavanagh and Murray 1996; Schoenefuss et al. 2024), dependably returning to one or more roosting sites (Cain et al. 2023), where they may deposit pellets over the long‐term (e.g., Avenant 2005; Bilney, Cooke, and White 2010). Initially, an owl pellet collection cannot be accurately dated, but once the roost is cleared, all subsequently deposited pellets may be reliably attributed to a time period (Schoenefuss et al. 2024).

The major component of rangeland (broadly the semi‐arid and arid zone, see Australian Government National Land And Water Resources Audit 2008) owl diets are small mammals (e.g., Bilney 2020; Debus, Ley, and Rose 2008; Schoenefuss et al. 2024). The technique relies on the widely accepted assumption that relative abundance of prey individuals found within a pellet collection is reflective of their abundance within the broader sampled community (Avenant 2005; Clulow et al. 2011; Heisler, Somers, and Poulin 2016; Kutt et al. 2021; McDonald, Burnett, and Robinson 2014; McDowell and Medlin 2009).

Owls effectively sample continuously (with one or more pellets regurgitated each night) despite changing environmental conditions, which may otherwise inhibit other monitoring methodologies. Thus, owls will potentially capture seasonal temporal shifts in mammal prey community structure (Avenant 2005; Debus, Ley, and Rose 2008; McDonald and Pavey 2014; Spencer, Newsome, and Dickman 2017; Torre et al. 2015). Indeed, owls may better reflect small mammal prey community composition than conventional trapping, particularly for trap‐shy, rare, cryptic and/or patchily distributed species (Heisler, Somers, and Poulin 2016; McDonald, Burnett, and Robinson 2014; Schoenefuss et al. 2024). Owl pellets may also yield large data sets, requiring a limited field time investment in remote areas that are otherwise expensive or difficult to survey (McDowell and Medlin 2009; van Strien et al. 2015).

One species to which owl pellet detection methodology may be readily applied is an Australian dasyurid, the Julia Creek dunnart, *Sminthopsis douglasi* . The species is listed as vulnerable under the Queensland *Nature Conservation Act* (1992) and the Australian *Environment Protection and Biodiversity Conservation Act* (1999). *Sminthopsis douglasi* is likely at risk of decline due to its limited distribution within an area subject to grazing, introduced predators and habitat modification by invasive weeds (Department of Environment and Resource Management 2009; Mifsud 1999). The largest of Australia's 19 dunnart species, *S. douglasi* is confined to the central and north‐west Queensland Mitchell Grass Downs, Gulf Plains and Desert Upland bioregions, most of which falls outside of protected areas (Baker and Gynther 2023; Kutt 2003). Indirect evidence suggests the species is nowhere abundant, likely occurring patchily in both time and space (Bakker, Patterson, et al. 2024; Mifsud 1999). Much of the habitat across the *S. douglasi* distributional range is thought to have declined in suitability due to increased grazing pressures and prickly acacia ( *Vachellia nilotica* ) densities (Smith et al. 2007). Notable exceptions include stock routes, road reserves and two national parks (Bladensburg and Moorrinya) (Smith et al. 2007). These various sites, and Toorak Station in Queensland's north‐west, have been identified as key management locations to ensure the long‐term protection of the species (Department of Environment and Resource Management 2009).

Described by Archer (1979) from only four museum specimens, *S. douglasi* was presumed extinct prior to 1992, when it was rediscovered as part of an extensive monitoring program (Woolley 1992). Bone collections, carcasses, owl pellet remains, predator gut contents and live trapping have all been used to corroborate species presence records (Kutt 2003; Mifsud 1999; Woolley 1992, 2010). *Sminthopsis douglasi* is notoriously difficult to detect (Bakker, Schoenefuss, et al. 2024), with trapping success highly variable and often below 1% (e.g., Baker, 2013; Bakker, Patterson, et al. 2024; Mifsud 1999, 2000, 2001b; Woolley 2016). The species has only been sporadically monitored over the last 25 years (e.g., Bakker, Patterson, et al. 2024; Kutt 2003; Woolley 2010, 2016). In some cases, publication of records occurred upwards of 10 years after the research was undertaken (e.g., Woolley 2010). Thus, population information is very limited and does not necessarily reflect the present‐day abundance of the species. Moreover, the total number of capture records is low, so distribution estimations may not represent fine‐scale spatial and temporal occurrence patterns (Kutt 2003). Flooding during 2019 (Barry 2019) and rodent plagues during 2023 and 2024 (Gall 2023; Paterson 2024) may have impacted inadequately monitored populations. Furthermore, existing studies are foundational (e.g., Mifsud 1999; Woolley 2010) and do not consider temporal dynamics, interactions with other species or potential range expansions. Indeed, prior to the live trapping study of Bakker, Patterson, et al. (2024) that estimated population density of the species at Bladensburg National Park, Kutt (2003) was the most recently published population data available for *S. douglasi*. Thus, there is a clear need for reassessment of *S. douglasi* populations at more locations.

Therefore, we aimed to assess changes in terrestrial small mammal community structure over time using eastern barn owl ( *T. javanica delicatula* ) pellet content analysis, with a focus on the threatened Julia Creek dunnart ( *S. douglasi* ) at Toorak. Present‐day collections encompassed an ongoing long‐haired rat ( *Rattus villosissimus* ) plague (Gall 2023; Paterson 2024) and permitted valuable insights into the broader community dynamics at the location and how this may impact *S. douglasi* populations. The present‐day collections were also compared to a published historical owl pellet dataset collected from Toorak between 1994 and 2001 (Woolley 2010), with an aim to inform directions for future management of the species at this site.

## 2 Methods

### 2.1 Study Area

**FIGURE 1:**
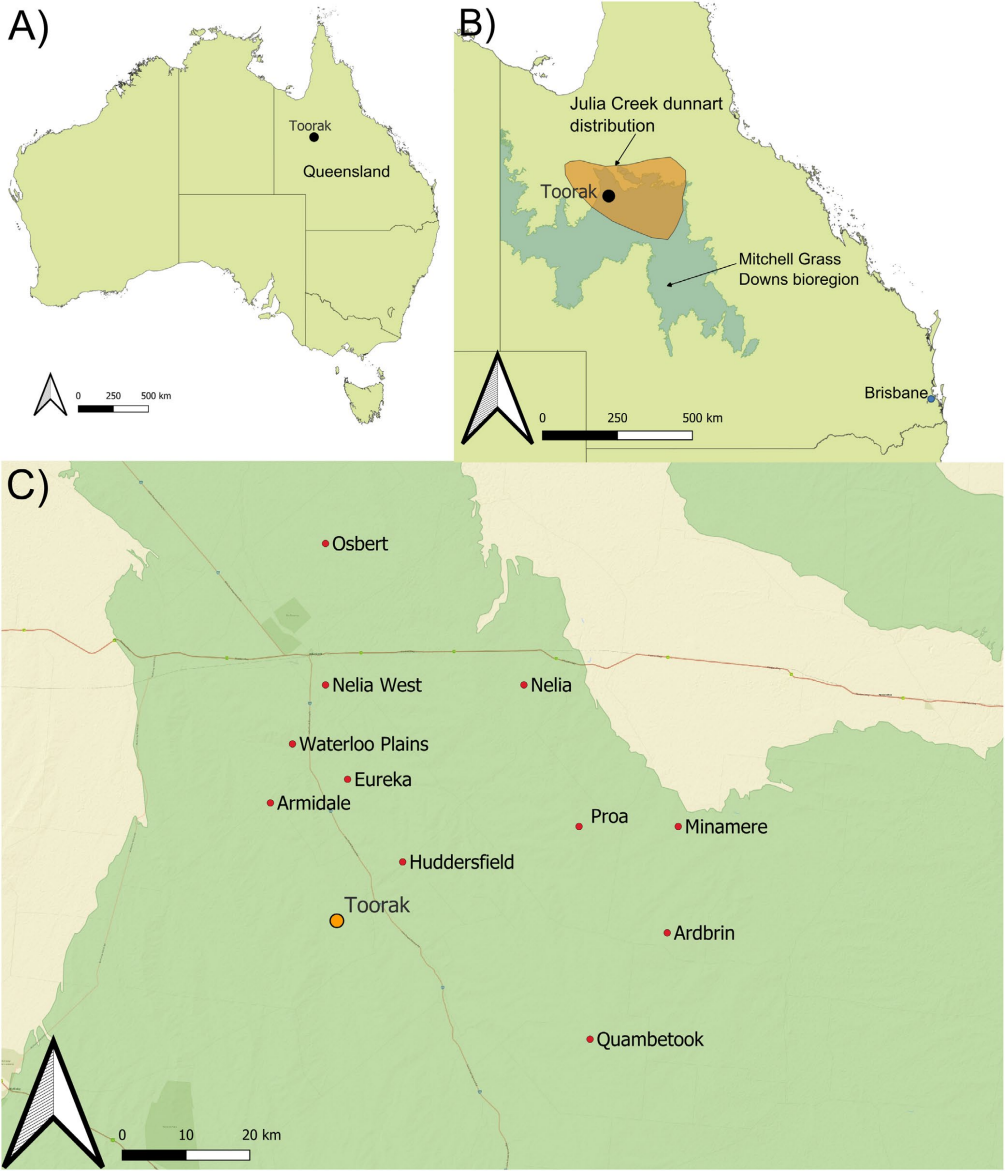
(A) Location of Toorak in reference to Australia. (B) Location of Toorak with reference to the state of Queensland. The orange shaded area represents an approximate Julia Creek dunnart distribution (Marsh et al. 2022). The light blue shaded area represents the Mitchell Grass Downs bioregion. (C) Location of eastern barn owl pellet collections assessed in this study. Toorak is the site of present‐day collections and the historic dataset taken from Woolley (2010). Other locations are owl pellet sites assessed by Woolley (2010) used in probability of detection analysis. The green shading represents the Mitchell Grass Downs bioregion.

Owl pellet collections were made at a single site: an abandoned woolshed at Toorak station in north‐west Queensland, Australia (21.03290S, 141.79958E, Figure 1). Toorak is located ~40 km south of Julia Creek and lies within the semi‐arid region of the Mitchell Grass Downs bioregion. Average annual rainfall is ~471 mm (at Julia Creek Airport, the closest weather station for Toorak; Australian Government Bureau of Meteorology 2024), supporting largely treeless grasslands on cracking clay soils, dominated by Mitchell grass (*Astrebla* spp.) and Flinders grass (*Iseilema* spp.) (White et al. 2014). Land use in the surrounding area is primarily pastoral on privately‐owned allotments (Australian Government National Land And Water Resources Audit 2008). Toorak was a long‐term livestock and vegetation research station prior to 2012, but it is now a privately‐owned cattle station (Bird 1989; Tapp 2012). *Sminthopsis douglasi* has a history of occurrence at Toorak, having been successfully live trapped (three individuals, 27,900 trap nights: Mifsud 1999), found in feral domestic cat ( *Felis catus* ) and European red fox ( *Vulpes vulpes* ) guts (Mifsud and Woolley 2012), and in barn owl pellet collections (13 individuals, 1994–2001: Woolley 2010).

Toorak has been identified as an important management location for the long‐term persistence of *S. douglasi* , with the national recovery plan recommending continued monitoring of the species at this location (Department of Environment and Resource Management 2009). Toorak was also a location used in the owl pellet analysis performed by Woolley (2010), and hence, this will serve as a comparative historical dataset in the present study. Other owl pellet locations used in probability of detection analyses were taken from Woolley (2010) (Figure 1C).

### 2.2 Owl Pellet Collections

This study was conducted under the auspices of Queensland Department of Environment, Science and Innovation (DESI) Permit P‐PTUKI‐100171210 and QUT Research Ethics Permit 5154.

Four separate collections were made, consisting of an undated initial collection and three dated collections, totalling 12 months of monitoring. Collection one (collected 13/04/2023, 280 pellets), may consist of up to a presumed approximate 5 years' worth of pellets (Kutt et al. 2021) and henceforth will be referred to as the Initial collection. The Mitchell Grass Downs broadly exhibit two seasons, a wet Summer (October to March) and a dry Winter (April to September) (Orr and Phelps 2013). The remaining collections made in the present research broadly encompass these seasons. Collection two (collected 18/07/2023, 156 pellets) and collection three (collected 02/10/2023, 113 pellets), when combined, represent ~6 months of Winter and henceforth will be referred to as the Winter collection. Collection four (collected 20/04/2024, 157 pellets) represents ~6 months of Summer and henceforth will be referred to as the Summer collection. Both the Winter and Summer collections encompassed a long‐haired rat ( *R. villosissimus* ) plague, which was potentially ongoing in the second half of 2024 (Gall 2023; Paterson 2024).

Only pellets deemed whole (i.e., those that maintained structural integrity and/or comprised large pellet fragments readily attributable to a single pellet), were used in further analysis. Nevertheless, each collection contained marked amounts of small fragmented, disintegrated or loose pellet material, which at times contained prey craniodental remains. The contents of the loose material were identified but not used in further formal statistical analysis as they could not be attributed to a discrete pellet.

### 2.3 Owl Pellet Dissection

Precautionary Q fever vaccinations were administered to personnel handling the pellets prior to commencement of lab work (NSW Health 2024). Pellets were stored dry, in bags, at room temperature and only with pellets representing the same collection. Owl pellet dissection was performed in a biosafety cabinet (reverse laminar airflow) with appropriate PPE (labcoat and gloves), to minimise potential exposure of lab personnel to fine particles.

Pellets were teased apart by hand. All identifiable craniodental material was separated from unidentifiable postcranial skeletal material and fur. Care was taken to ensure all craniodental material was separated from all other pellet content. Craniodental material was preliminarily identified to family level. Both identifiable and unidentifiable remains were stored in QUT freezers at −20°C for a minimum of 10 days prior to further analysis at the Queensland Museum, to kill invertebrates that may otherwise have infested the museum collection.

### 2.4 Species Identification

The focus in the present study was mammal prey, but all vertebrates in pellets were identified to the lowest possible taxonomic level. No attempt was made to identify or enumerate invertebrate material in the pellets.

A potential small mammal species list was compiled for Toorak based on species distribution maps and habitat information retrieved from Baker and Gynther (2023) and Van Dyck, Gynther, and Baker (2013). Exemplar craniodental material was assembled for all potential mammal species using the Queensland Museum registered mammal skull collection. Comparative museum material was also assessed for birds, reptiles and frogs that may occur in the region.

**FIGURE 2:**
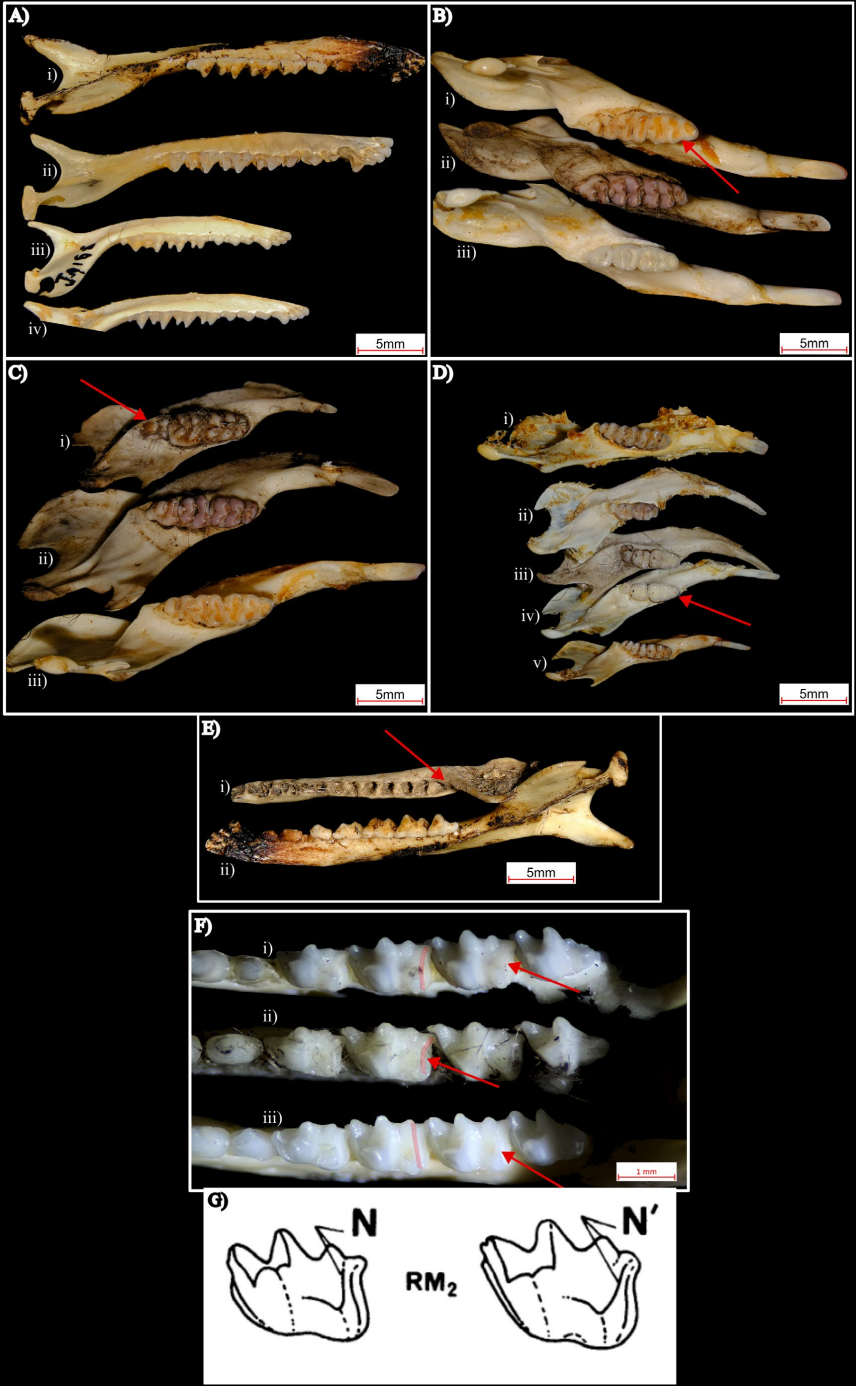
Examples of craniodental features used to distinguish between features of small mammal species found in eastern barn owl pellets. Specimens used are from the Queensland Museum reference collection unless stated otherwise (pellet). Part (A) compares *Sminthopsis* mandibles, (i) pellet *Sminthopsis douglasi* , (ii) *Sminthopsis douglasi* , (iii) *Sminthopsis macroura*, (iv) *Sminthopsis crassicaudata* . Part (B) compares *Rattus* mandibles, (i) *Rattus villosissimus* , (ii) pellet *Rattus villosissimus* , (iii) *Rattus rattus* . Part (C) compares juvenile dental features in *Rattus*, (i) pellet juvenile *Rattus villosissimus* , (ii) adult *Rattus villosissimus* , (iii) Adult *Rattus villosissimus* . Part (D) compares small rodent dental features, (i) *Pseudomys delicatulus* , (ii) *Leggadina lakedownensis* , (iii) pellet *Leggadina forresti* , (iv) *Leggadina forresti* , (v) *Pseudomys delicatulus* . Part (E) compares tooth row length and other mandible features in (i) pellet juvenile *Sminthopsis douglasi* and (ii) pellet adult *Sminthopsis douglasi* . Part (F) compares hypocristid‐entoconid condition between (i) *Sminthopsis macroura* , (ii) pellet *Sminthopsis macroura* , (iii) *Sminthopsis crassicaudata* . Part (G) visualises the hypocristid‐entoconid condition used for *Sminthopsis crassicaudata* (N) and *Sminthopsis macroura* (N′) identification, *adapted from* Archer (1981). Images primarily taken with a Fujifilm X‐T5 camera and Fujinon XF 70–300 mm f/4–5.6 lens or a Zeiss Stemi 305 stereo microscope with attached Axiocam 208 colour microscopy camera. See Appendix 1 for further details about species identification.

Pellet‐based craniodental material was identified under a stereo microscope (Zeiss Stemi 305), with reference to the registered museum specimens, and using skull, mandible and dentition features described in Archer (1981) and Van Dyck, Gynther, and Baker (2013). The idiosyncratic nature of mammalian craniodental material enabled confident identification to species in most cases (Archer 1981). Identification of other groups, such as birds, was more conservative (sometimes to species, or if not genus or family) and relied primarily on comparison to museum reference skeletal specimens and expert assistance (Heather Janetzki, Queensland Museum). In most cases, skull pieces in owl pellets were highly fragmented and could not be readily used alone for identification purposes. Hence, most mammal species identifications were made using mandible and tooth characteristics, which in combination were highly consistent and idiosyncratic in the taxa examined (Figure 2). See Appendix 1 for more details.

The minimum number of individuals per pellet was recorded, based on the number of unique mandibles (e.g., two left mandibles and one right mandible was counted as two individuals) (Kutt et al. 2021). The number of individuals was corroborated where possible using other craniodental material (skulls). Time taken to both separate and identify material was recorded to the nearest 5 min.

### 2.5 Statistical Analyses

Analyses were performed in R (R Core Team 2024). Toorak was a location assessed by Woolley (2010), and it was used here as a comparative dataset, henceforth termed the Historic dataset. In Woolley (2010), Barn owl pellets were collected between 1994 and 2001, although primarily between 1998 and 2001, composed of varying collection sizes and grouped as a complete Historical Toorak collection. Analysis, unless stated otherwise, was performed on the four periods (Initial 2023, Winter 2023, Summer 2024 [from the present study] and Historic [from Woolley (2010)]).

Woolley (2010) did not provide data about juvenile/adult proportions, nor individual counts of taxa other than for mammals. Therefore, for comparative analysis with the Historic dataset, data from the present study matched the taxonomy used by Woolley (2010) (e.g., although identified to species or family in present‐day collections, birds were grouped under the broad Class Aves when compared to the Historical collection).

Woolley (2010) classified all planigale individuals from Toorak as *Planigale ingrami* based on (as yet unpublished) observational data. However, in the present study, confident discrimination of planigale species from owl pellets was not possible. This was because of similarities in craniodental features of planigale species (Archer 1976; Blacket, Kemper, and Brandle 2008), the material found in pellets was often only mandibles or otherwise fragmented, and there are potentially overlapping distributions of two planigales at Toorak, *P. ingrami* and *Planigale tenuirostris* (Baker and Gynther 2023). Thus, all planigales were conservatively treated as *Planigale* spp. in our comparative analysis, and unlike other dasyurids, juvenile planigales could not be reliably discriminated from adults.

The number and proportion of pellets containing a species (irrespective of total pellet number), the proportion of the total number of individuals for each species and the average minimum number of prey items per pellet (based on the number of pellets containing identifiable vertebrate material, that is, removing those containing solely unidentifiable remains) were calculated.

Pairwise Chi‐squared analysis was performed to assess differences in species relative abundance between periods, using the *rstatix* R package (Kassambara 2023). This allowed a ‘post hoc‐like’ assessment of mammal community structure across the three assessed time periods, and to the Historical dataset (Schoenefuss et al. 2024). A pairwise Fisher's exact test was used where assumptions of Chi‐squared testing were not met (Fisher 1922). Correction for multiple comparisons followed the Holm‐Bonferroni method (Holm 1979).

Hill numbers (Chao et al. 2014; Hsieh, Ma, and Chao 2016) were used to assess differences in species richness and diversity between the four collections. Hill numbers (Hill Diversity) represent the effective number of species calculated from a shared equation of various orders (q), as per Chao et al. (2014). For species richness (q = 0), species are counted equally without regard to relative abundances (Roswell, Dushoff, and Winfree 2021). For Hill‐Shannon diversity (q = 1), individuals are counted equally and can be interpreted as the effective number of common species (Roswell, Dushoff, and Winfree 2021). Hill‐Simpson diversity (q = 2) can be interpreted as the effective number of dominant species (Chao et al. 2014; Colwell et al. 2012). Hill numbers were generated using the *iNEXT* R package (50 bootstrap replications) (Hsieh, Ma, and Chao 2016).

To assess the effectiveness of owl pellets as a small mammal sampling technique, probability of detection, including cumulative probability of detection curves, were generated based on McCallum (2005). Probability of detection for each species was calculated from the following formula:

$$\mathrm{pn}=1-{\left(1-\left(C/N\right)\right)}^{n}$$

Where pn is the cumulative probability of detection from a sample size of n, N is the total number of pellets assessed and C is the number of pellets containing the target species. A *pn* > 0.95 was chosen to represent a level for confident detection (based on α = 0.05). This was calculated for mammal species only. Additionally, probability of *S. douglasi* detection was calculated for various other sites (Figure 1A, > 3 *S. douglasi* individuals) assessed by Woolley (2010).

## 3 Results

The number of individual owls contributing to both the present day and historic (Woolley 2010) pellet collections is unknown, although a single owl was observed at the roost for both collection 1 (April 2023) and collection 2 (July 2023). A dead owl and two live owls were observed during collection 3 (October 2023), and two live owls were observed during collection 4 (April 2024).

A total of 1007 individual vertebrates were identified from 646 owl pellets, yielding an average of 1.56 individuals per pellet, with an additional 60 pellets containing only unidentifiable vertebrate postcranial material (Table 1), for a combined total of 706 pellets assessed. Approximately 70 additional vertebrate individuals were identified in loose pellet material from an unknown number of pellets (Table A1). The latter material was not readily attributable to individual prey items and was not included in further analyses. Nevertheless, it represented another 25 putative *S. douglasi* individuals, among other species.

**TABLE 1:** Individual prey items identified in the three assessed collections.

Species	Age class	Initial collection (collection 1 April 2023, 280 pellets)				Winter (collections 2 and 3, July and October 2023, 269 pellets)				Summer (collection 4 April 2024, 157 pellets)			
		No. pellets	% total pellets	No. ind	% ind	No. pellets	% total pellets	No. ind	% ind	No. pellets	% of total pellets	No. ind	% ind
Mammals													
*Sminthopsis douglasi*	Juvenile	30	10.71	36	8.20	18	6.69	19	4.45	4	2.55	4	1.99
	Adult	82	29.29	99	22.55	46	17.10	49	11.48	19	12.10	20	9.95
*Sminthopsis macroura*	Juvenile	7	2.50	9	2.05	5	1.86	6	1.41	1	0.64	1	0.50
	Adult	6	2.14	7	1.59	6	2.23	7	1.64	3	1.91	3	1.49
*Planigale* spp.	—	32	11.43	46	10.48	19	7.06	36	8.43	8	5.10	14	6.97
*Rattus villosissimus*	Juvenile	16	5.71	20	4.56	34	12.64	57	13.35	24	15.29	30	14.93
	Adult	83	29.64	86	19.59	79	29.37	93	21.78	79	50.32	85	42.29
*Leggadina forresti*	Juvenile	0	0	0	0	0	0	0	0	1	0.64	1	0.50
	Adult	2	0.71	2	0.46	2	0.74	2	0.47	3	1.91	3	1.49
Birds													
*Taeniopygia* sp.		24	8.57	51	11.62	26	9.67	42	9.84	3	1.91	4	1.99
*Artamus* sp.		16	5.71	17	3.87	13	4.83	15	3.51	3	1.91	3	1.49
*Mirafra javanica*		10	3.57	11	2.51	10	3.72	10	2.34	2	1.27	2	1.00
*Melopsittacus undulatus*		5	1.79	6	1.37	5	1.86	7	1.64	1	0.64	1	0.50
*Anthus australis*		2	0.71	2	0.46	0	0	0	0	2	1.27	2	1.00
Class Aves		23	8.21	26	5.92	43	15.99	51	11.94	9	5.73	9	4.48
Amphibians													
Order Anura		3	1.07	4	0.91	4	1.49	4	0.94	0	0	0	0
Reptiles													
Family Gekkonidae		1	0.36	1	0.23	1	0.37	1	0.23	0	0	0	0
Family Agamidae		0	0.00	0	0	3	1.12	3	0.70	0	0	0	0
Unidentifiable postcranial		16	5.71	16	3.64	25	9.29	25	5.85	19	12.10	19	9.45

*Note:* No. pellets represent the total number of pellets containing an individual irrespective of the number of individuals within said pellet. % total of pellets represents the percentage of pellets containing individuals of that species. No. ind represents the number of unique individuals found in all pellets from that collection, while % ind represents No. ind as a proportion of the total number of individuals of all species from that collection.

With no prior experience, it took ~49 h for pellet dissection and a further ~67 h for identification, for a combined average of ~7 min per individual or ~10 min per pellet (minutes rounded up). Of the 1007 individual vertebrates, terrestrial mammals were most frequent (72.99%, 735 individuals), followed by birds (25.72%, 259 individuals), frogs (0.79%, 8 individuals) and finally reptiles (0.50%, 5 individuals). Considering the three collections separately, mammals comprised 69.48%, 63.01% and 80.10% of all individuals for Initial, Winter and Summer, respectively, followed by birds (25.74%, 29.27%, 10.45%), frogs (0.91%, 0.94%, 0%) and reptiles (0.23%, 0.93%, 0%). Unidentifiable postcranial material increased between Initial (3.64%), Winter (5.85%) and Summer (9.45%) collections. No arboreal or aerial mammals were recorded in the pellets.

**FIGURE 3:**
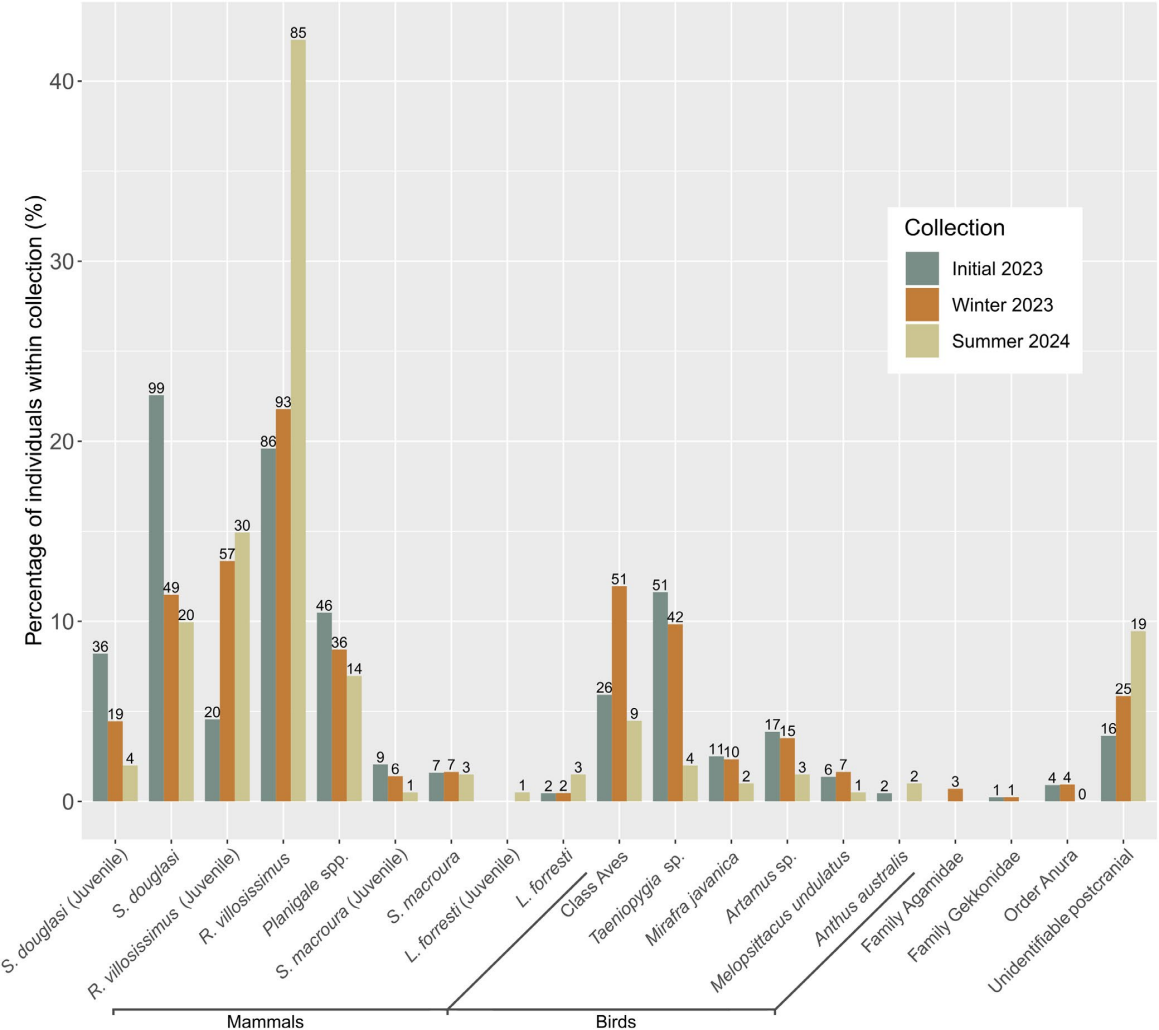
Variation in the percentage of individuals within each collection. Values above each bar represent the number of individuals of each species grouping. For mammals, species groupings are split between adult and juvenile where possible. Otherwise, all other groupings encompass both adults and juveniles.

All dasyurid marsupials decreased between Initial, Winter and Summer pellet collections, while rodent species showed marked increases (Figure 3). Birds generally showed a similar decrease, apart from unidentified birds (Class Aves), which increased during Winter 2023. Juvenile *R. villosissimus* maintained similar proportions between Summer and Winter, compared to adults, which markedly increased in Summer. Juvenile *S. douglasi* and *Sminthopsis macroura* showed marked decreases across all collections comparable to trends observed in their adult counterparts (Figure 3).

### 3.1 Comparisons to Historic Dataset

**FIGURE 4:**
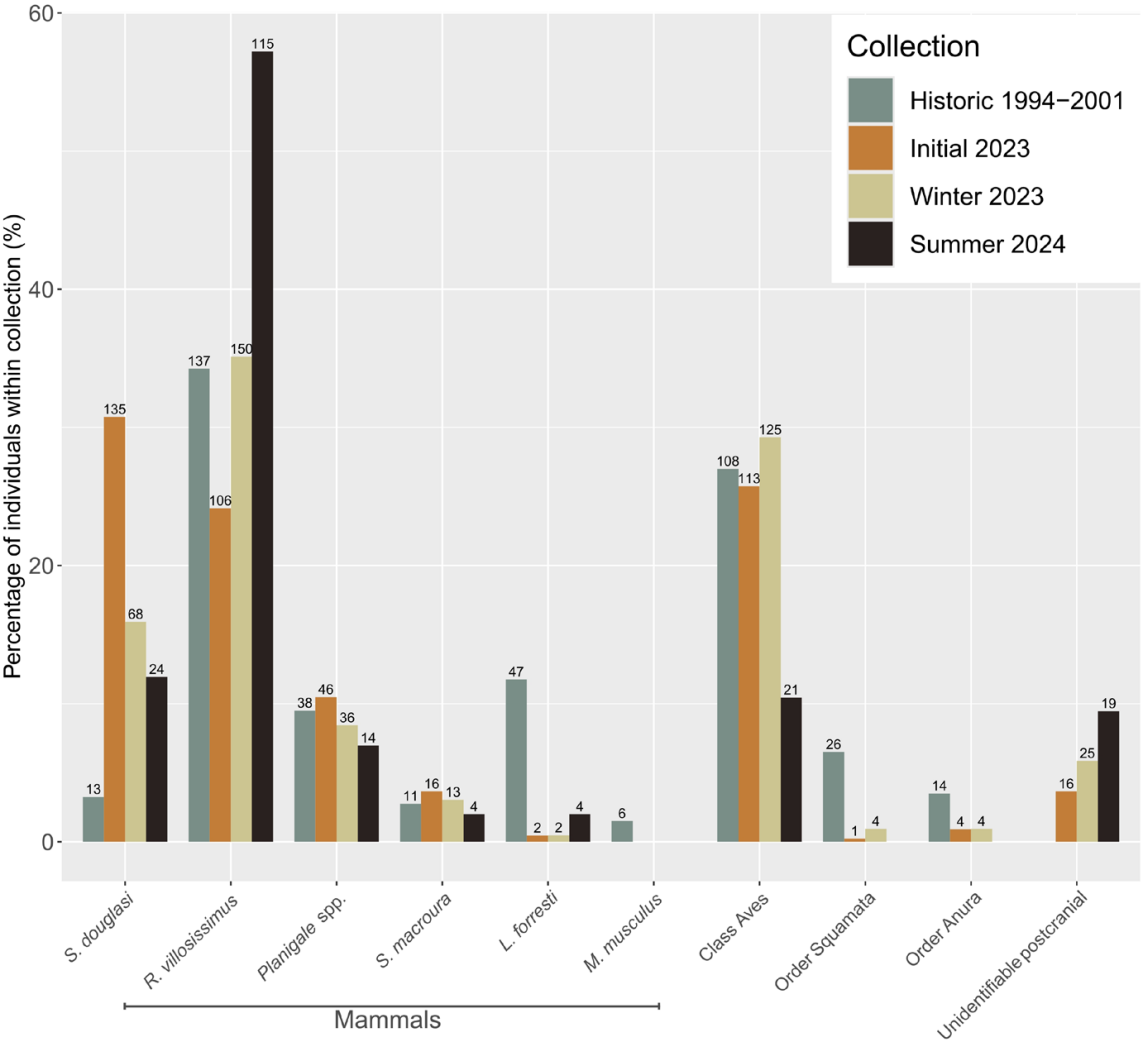
Variation in the percentage of individuals within each collection assessed, including Historic (Woolley 2010). Values above each bar represent the number of individuals of each species grouping.

The mammal community structure for the Historic dataset was notably different compared to all three present‐day collections (Table 2, Figure 4). For *S. douglasi* , proportion of individuals was significantly different for all pairwise dataset comparisons other than Winter (15.92%) versus Summer (11.94%) (*p*‐value = 0.23, *χ* ^2^ = 1.43) (Table 2). The proportion of *S. douglasi* individuals was lowest in the Historic collection (3.25%), markedly greater in all present‐day collections and highest in the Initial collection (30.75%). For *R. villosissimus* , there was no significant difference in the proportion of individuals for Winter (35.13%) versus Historic (34.25%) (*p*‐value = 0.85, *χ* ^2^ = 0.04). All other pairwise comparisons were significantly different (Table 2). *Rattus villosissimus* individual proportions were highest in Summer (57.21%), and lowest in Initial (24.15%), showing an overall increase from 2023 to 2024. There was no significant difference for *S. macroura* and *Planigale* spp. individual proportions between all collections. The *Leggadina forresti* individual proportion in the Historic collection (11.75%) was significantly higher than all present‐day collections. This rodent species was rarely observed in present‐day collections, with the highest proportion (1.99%) found in Summer. *Mus musculus* was not found in any present‐day collections.

**TABLE 2:** Results of pairwise Chi‐squared and Fisher tests comparing all four analysed collections.

	Species	*Sminthopsis douglasi*	*Sminthopsis macroura*	*Planigale* spp.	*Rattus villosissimus*	*Leggadina forresti*	*Mus musculus*	Aves spp.	Order Squamata	Order Anura	Unidentifiable postcranial
	*df*	1	1	1	1	n/a	n/a	1	n/a	n/a	1
	Test	Chi‐squared	Chi‐squared	Chi‐squared	Chi‐squared	Fisher	Fisher	Chi‐squared	Fisher	Fisher	Chi‐squared
Initial versus Winter	*n*	866	866	866	866	866	866	866	866	866	866
	*χ* ^2^	25.69	0.09	0.83	12.02	n/a	n/a	1.18	n/a	n/a	1.88
	*p*	< 0.01	1	1	< 0.01	1	1	0.83	0.63	1	0.28
	Significance	****	ns	ns	**	ns	ns	ns	ns	ns	ns
Initial versus Summer	*n*	640	640	640	640	640	640	640	640	640	640
	*χ* ^2^	25.13	0.76	1.61	65.23	n/a	n/a	18.57	n/a	n/a	7.91
	*p*	< 0.01	1	1	< 0.01	0.24	1	< 0.01	1	0.94	0.01
	Significance	****	ns	ns	****	ns	ns	****	ns	ns	*
Winter versus Summer	*n*	628	628	628	628	628	628	628	628	628	628
	*χ* ^2^	1.43	0.25	0.23	26.43	n/a	n/a	26.10	n/a	n/a	2.19
	*p*	0.23	1	1	< 0.01	0.24	1	< 0.01	0.63	0.94	0.28
	Significance	ns	ns	ns	****	ns	ns	****	ns	ns	ns
Initial versus Historic	*n*	839	839	839	839	839	839	839	839	839	839
	*χ* ^2^	107.07	0.29	0.13	9.90	n/a	n/a	0.11	n/a	n/a	12.98
	*p*	< 0.01	1	1	< 0.01	< 0.01	0.068	1	< 0.01	0.07	< 0.01
	Significance	****	ns	ns	**	****	ns	ns	****	ns	**
Winter versus Historic	*n*	827	827	827	827	827	827	827	827	827	827
	*χ* ^2^	36.13	0.002	0.17	0.04	n/a	n/a	0.42	n/a	n/a	22.19
	*p*	< 0.01	1	1	0.85	< 0.01	0.068	1	< 0.01	0.07	< 0.01
	Significance	****	ns	ns	ns	****	ns	ns	***	ns	****
Summer versus Historic	*n*	601	601	601	601	601	601	601	601	601	601
	*χ* ^2^	16.02	0.08	0.79	28.04	n/a	n/a	20.77	n/a	n/a	36.02
	*p*	< 0.01	1	1	< 0.01	< 0.01	0.744	< 0.01	< 0.01	0.02	< 0.01
	Significance	***	ns	ns	****	****	ns	****	***	*	****

*Note:* The number of *'s represents the significance level (*p ≤ 0.05, **p ≤ 0.01, ***p ≤ 0.001, ****p ≤ 0.0001) whereas ns represents a non‐significant result.

Overall, bird individual proportions were comparable across Historic (27%), Initial (25.74%) and Winter (29.27%) collections, with significant differences driven by a decrease in Summer (10.45%) (Table 2). Individual proportions of both lizards and frogs were significantly higher in the Historic collection (6.5%, 3.5%, respectively) (Table 2), and these taxa were rarely found in the present‐day collections.

### 3.2 Species Diversity

Small mammal diversity varied across all assessed pellet collections. Species richness was the same across all present‐day collections (five species), while the Historic collection was higher at 6 species due to the presence of *M. musculus* . For Hill‐Shannon diversity, Summer had the lowest diversity (2.54), likely due to the dominance of *R. villosissimus* . Historic was the most diverse (3.74), followed by Initial (3.35) and Winter (3.11), indicative of a more even spread of individual proportions across taxa groups. Hill‐Simpson diversity had comparable results to Hill‐Shannon diversity, with differences primarily driven by the extent of dominance of *R. villosissimus* . Initial was the most diverse (2.94), followed by Historic (2.81), Winter (2.55) and Summer (1.86).

### 3.3 Probability of Detection

**FIGURE 5:**
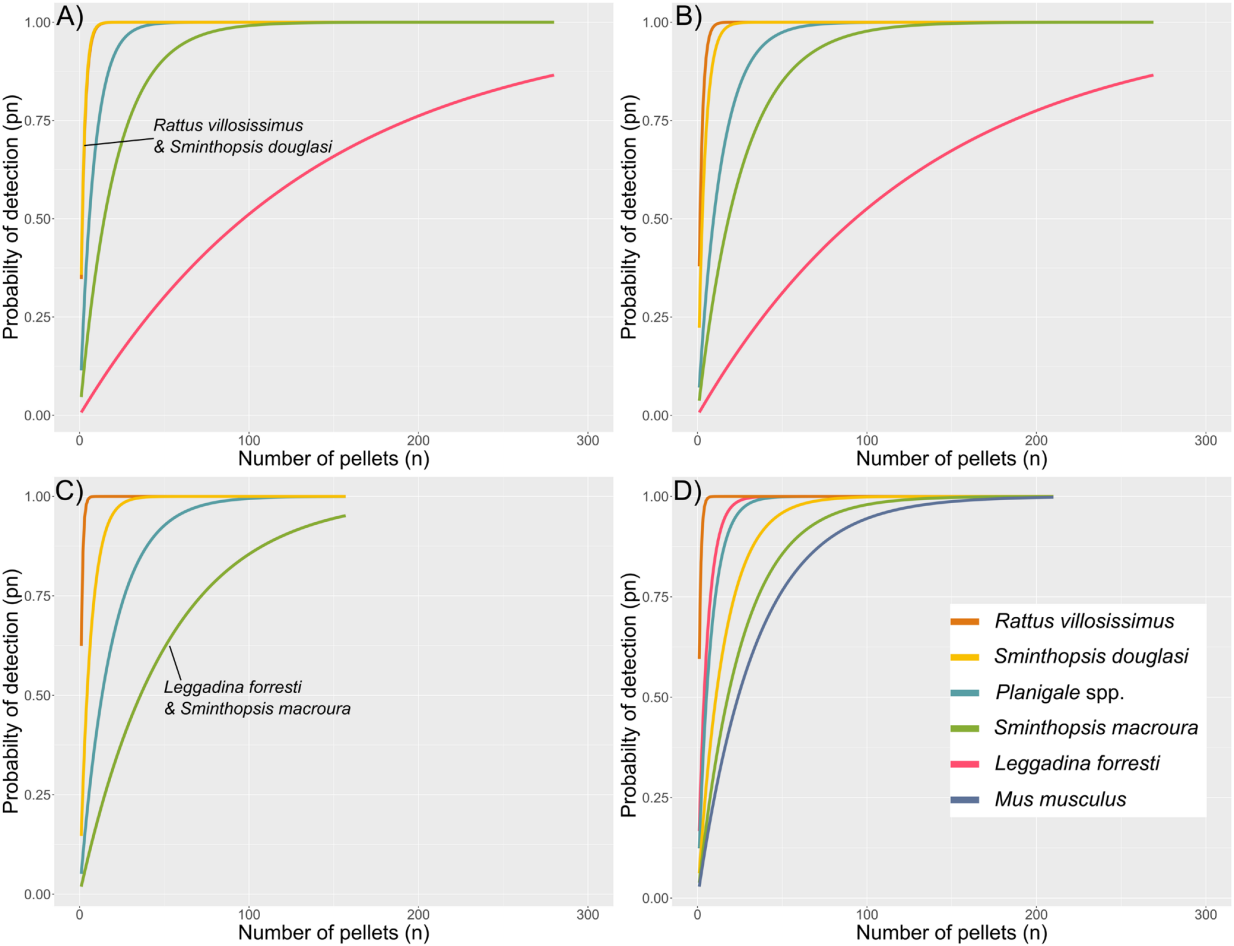
Mammal prey species cumulative probability of detection (*pn*) versus number of owl pellets assessed (*n*) for (A) Initial, (B) Winter, (C) Summer and (D) Historic, collections. Curves start at 1 pellet assessed, as assessing 0 pellets is unrealistic in application. Labels signify overlapping probability of detection curves.

Cumulative probability of detection varied between species, but in the majority of cases a *pn* > 0.95 was reached within 160 pellets examined (Figure 5). Cases where *pn* > 0.95 was not reached was driven by a low sample size, primarily for *M. musculus* and *L. forresti*. For *S. douglasi* , a *pn* > 0.95 was reached for all cases in under 50 pellets. For the present‐day collections, *pn* > 0.95 was reached in under 20 pellets in all collections, with only seven pellets required to confidently detect *S. douglasi* in the Initial collection. *Rattus villosissimus* was consistently detected (*pn* > 95) in under 10 pellets across all assessed collections, with a faster detection rate in Summer (four pellets).

**FIGURE 6:**
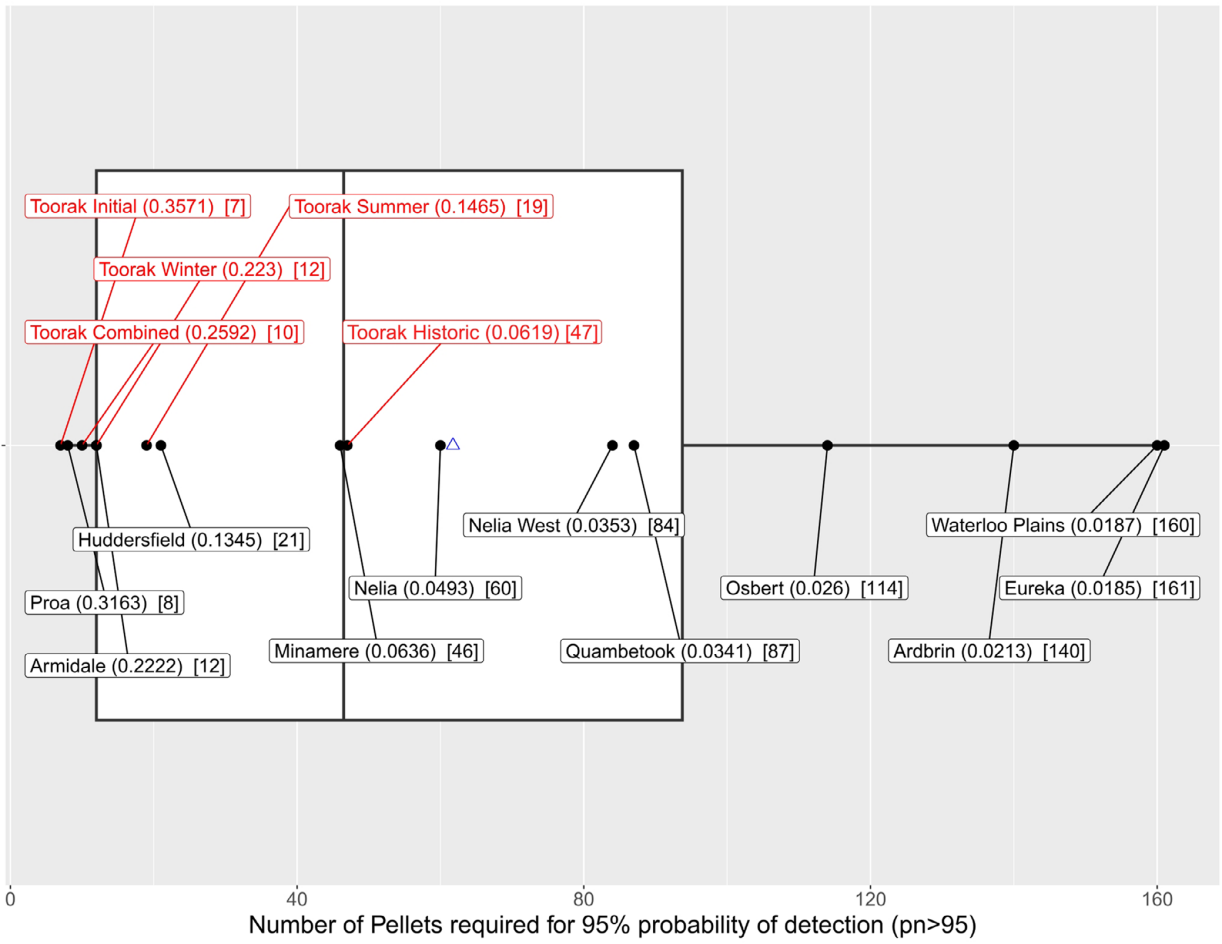
Cumulative probability of detection for *Sminthopsis douglasi* as a box plot showing comparison of Toorak (red text; present collections, Historic and combined) versus other sites (black text) assessed by Woolley (2010) where number of *S. douglasi* individuals was > 3. Numbers in () represent a decimal probability of detection at 1 pellet, while numbers in [] represent the number of pellets required for a *pn* > 95. The blue triangle indicates the mean number of pellets for all collections combined. The white box represents the interquartile range and the vertical line the median number of pellets.

Considering only *S. douglasi* at Toorak, the number of pellets required to reach a confident cumulative probability of detection was very low across all assessed collections (Figure 5). When considering all pellet locations in Woolley (2010) where > 3 *S. douglasi* individuals were found (Figure 6), the present‐day Toorak collection was not an outlier for *S. douglasi* detection rates. Cumulative probability of detection (*pn* > 95) for Proa (8 pellets), Armidale (12 pellets) and Huddersfield (21 pellets) were all comparable to present‐day collections, while Minamere (46 pellets) and Nelia (60 pellets) were comparable to the Historic collection (Figure 6 and see also Figure 1C for site localities). The most pellets required for confident detection of *S. douglasi* was 161 (Eureka).

## 4 Discussion

The present study primarily aimed to assess changes in small mammal community structure over time using eastern barn owl ( *T. javanica delicatula* ) pellet content analysis, with a focus on the threatened Julia Creek dunnart ( *S. douglasi* ), at Toorak, a key management site for the long‐term persistence of the species. We demonstrated that not only was *S. douglasi* a key prey item for owls at Toorak during all time periods, but that the species could be reliably detected using owl pellets even during an ongoing native long‐haired rat ( *R. villosissimus* ) plague. We have undertaken the first reassessment of the terrestrial small mammal community at Toorak based on owl pellets since Woolley (2010), where the historical data was collected upwards of 20 years ago, and the first assessment since Toorak has transitioned from a research station to a commercial cattle station. Thus, we have provided a much‐needed update to the population status of *S. douglasi* at a key conservation location for the species.

### 4.1 *Sminthopsis douglasi*

Despite declines in relative abundance at Toorak within present‐day collections (2023–2024), *S. douglasi* had individual proportions significantly higher than those observed by Woolley (2010) for all present‐day collections, peaking at almost tenfold higher (Initial, 30.75% versus Historic, 3.25%). This finding supports the identification of Toorak as a key location for the long‐term persistence of the species (Department of Environment and Resource Management 2009; Smith et al. 2007). *Sminthopsis douglasi* is potentially more abundant at Toorak than previously described (Mifsud 1999; Woolley 2010). Additionally, flooding throughout the Julia Creek region during 2019 (Barry 2019) and the changed land use at Toorak has apparently not impacted the persistence of the population over time. If further monitoring, via owl pellets, live trapping and/or cameras (Bakker, Schoenefuss, et al. 2024) corroborates a large and stable population of *S. douglasi* , the protection of Toorak via a voluntary conservation agreement should be pursued as per Objective 2 of the National Recovery Plan (Department of Environment and Resource Management 2009).

Owl pellet analysis ultimately relies on the behavioural preferences of individual owls for how they select prey, particularly as multiple owls may contribute to a single pellet collection (Schoenefuss et al. 2024). The number of individual barn owls contributing to the various pellet collections assessed here is unknown, although based on observations made at the time of pellet collection (present day), we may expect between two and three different individuals contributing to this roosting site. Based on the rate of pellet deposition, there may be a secondary roost site (assuming at least one pellet deposited every 24 h). The number of owls contributing to the Historic collection is unknown (Woolley 2010).

It is widely accepted that owl diet preference, and hence the relative abundance of prey items found in pellets, is reflective of species abundance within the surrounding ecosystem (e.g., Andrade, de Menezes, and Monjeau 2016; Bilney 2020; Clulow et al. 2011; Debus and Rose 2003; Debus, Rose, and Harris 1999; Heisler, Somers, and Poulin 2016; Heywood and Pavey 2002; Kutt et al. 2021; Schoenefuss et al. 2024; Torre et al. 2015; Woolley 2010). Despite this, a legitimate concern is that owls may have a tendency for bias towards certain prey items (Bilney, Cooke, and White 2010; Pavey, Gorman, and Heywood 2008; Smith 1984), particularly for large, energetically profitable meals (Yom‐Tov and Wool 1997). This bias may also extend to species which are more (or less) accessible to owl predation, due to behavioural tendencies (e.g., diurnal species are intrinsically less likely to be predated by a nocturnal owl) or ecological niche use (e.g., occupation of complex habitats may inhibit an owl hunting). The present probability of detection analysis indicated detection of *S. douglasi* within just 20 pellets for all present‐day collections, suggesting the species occurs in high abundance at this location. It is plausible that the eastern barn owls at Toorak, for reasons other than solely the abundance of *S. douglasi* at the location, were preferentially selecting and thus overestimating relative abundance of the species as prey. However, it is notable that multiple other locations assessed over time by Woolley (2010) were comparable to the cumulative probability of detection rates observed at Toorak (Figure 6), suggesting that generally owl pellets are a highly effective tool for assessing the presence of *S. douglasi* . Furthermore, despite the preference of *S. douglasi* for cracks in the soil and tussock grass cover (Mifsud 1999), which could plausibly decrease predation rates by owls, the species was present in high abundance in the owls' diet.

Based on the *S. douglasi* cumulative probability of detection analysis, the maximum number of pellets required for confident detection was 161. By extrapolation, a conservative minimum of ~200 pellets are suggested as the collection size needed to detect the presence of a *S. douglasi* population regardless of the species relative abundance within the assessed prey community. Similar owl pellet studies indicate a comparable pellet number required to maximise species richness sampling, although this may vary dependent on the average number of individual prey items per pellet (e.g., Jiménez‐Nájar et al. 2021; Purger and Szép 2022; Schoenefuss et al. 2024).

The present study also highlights the utility of owl pellet analysis to maintain high detection rates of *S. douglasi* despite changing community structure at Toorak, particularly in relation to a native *R. villosissimus* plague. In general, live trapping success for *S. douglasi* is highly variable and often below 1% (e.g., Baker, 2013; Bakker, Patterson, et al. 2024; Mifsud 1999, 2000, 2001a, 2001b; Woolley 2016). During rodent plagues, trapping for *S. douglasi* is highly ineffective, as *R. villosissimus* saturate traps, often resulting in no captures of *S. douglasi* despite confirmation of presence during the same period (e.g., via camera traps; Bakker, Schoenefuss, et al. 2024). In contrast, here only 19 pellets were required for detection of the target species through the height of the assessed *R. villosissimus* plague (Summer 2024), despite a significant decrease in observed *S. douglasi* relative abundance as prey during that period. Therefore, owl pellet content analysis may prove highly effective at maintaining *S. douglasi* detections despite changing environmental conditions, which may otherwise hinder a traditional live trapping focused monitoring regime.

### 4.2 The Influence of *Rattus villosissimus*

*Rattus villosissimus* relative abundance in pellets significantly differed between all assessed collections (other than Historic versus Winter 2023), being the highest in Summer 2024 (57.21%) and the lowest in the Initial collection in 2023 (24.15%). *Rattus villosissimus* is a key prey item for eastern barn owls (Clulow et al. 2011; Debus and Tsang 2023; Greenville, Wardle, and Dickman 2013; Morton 1975), and its presence in all assessed collections was expected. High annual rainfall was experienced in the region during late‐2022 to early‐2023 (Australian Government Bureau of Meteorology 2024). *Rattus villosissimus* has known irruptive population dynamics subsequent to rainfall (Carstairs 1976; D'Souza et al. 2013; Plomley 1972; Predavec and Dickman 1994; Tatler et al. 2019). Thus, increases in *R. villosissimus* relative abundance observed in Winter 2023 and Summer 2024 collections were likely reflective of a rainfall driven resource pulse throughout the region resulting in a rodent plague. Such plagues may be widespread and last for multiple years (Carstairs 1976).

In the present study, reports of *R. villosissimus* plagues throughout the Toorak‐Julia Creek region started during early 2023 and were common in local media from mid‐2023 (Gall 2023). The plague was also observed in live and camera trapping undertaken at Bladensburg National Park (approx. 200 km south‐east of Toorak; Bakker, Schoenefuss, et al. 2024) and ongoing trapping and camera deployments at Proa and Wensley (~40 km north‐east of Toorak; A.M. Baker and Southern Gulf NRM, unpublished). Indeed, the rodent plague continued into the second half of 2024 (October 2024; Paterson 2024). Given the date of the Initial collection (pellets likely primarily represent a period pre‐2023), it is assumed that this collection does not represent a plague period and is rather representative of a ‘baseline’ community structure. If so, the Winter collection may represent an intensifying rodent plague at Toorak, while Summer represents the plague in force. Owl pellets have demonstrated the buildup of *R. villosissimus* relative abundance across this period. The high relative abundance of *R. villosissimus* in the Initial collection, deemed a likely non‐plague period, may further support the suggestion that Toorak (and by extension the broader Julia Creek region) may serve as a refugium for the species during non‐plague periods (Woolley 2010). The similarities in *R. villosissimus* proportions between the Historic collection and Winter 2023 suggest that the Toorak pellets assessed by Woolley (2010), which were primarily collected between 1998 and 2001, may encompass an intensifying plague period. It is noteworthy that Mifsud (1999) suggested the possibility of increasing *R. villosissimus* abundance at Proa (~40 km north‐east of Toorak) during mid‐1998 trapping.

### 4.3 Other Changes in Vertebrate Community Structure at Toorak

Both *Planigale* spp. and *S. macroura* had comparable individual proportions in the present‐day analyses to Woolley (2010), although both species did decline as owl prey items throughout 2023–2024, albeit not significantly. Dickman and Pavey (2023) describe rangeland dasyurids as ‘evaders’, whose adaptations allow persistence despite extreme climatic variability. Many dasyurids do not respond in severe boom‐bust cycles and are rather limited by local conditions and life‐history constraints (Dickman et al. 2001; Dickman and Pavey 2023; Greenville, Wardle, and Dickman 2012; Greenville et al. 2016). *Sminthopsis crassicaudata* was not found in the present study despite plausible occurrence at the study site (Baker and Gynther 2023), a finding consistent with Woolley (2010).

In the present study, *S. douglasi, Planigale* spp. and *S. macroura* represented a combined 44.87% of all individual prey items during the non‐plague period (Initial). This finding corroborates the work of Heywood and Pavey (2002), who suggested that dasyurids may be important prey for owls outside rodent plague periods, although it is notable that rodents also formed a key component of owl diet during this period (Initial). While it is not universal (Dickman et al. 2001; Greenville et al. 2018; Pavey, Nano, and Waltert 2020), some dasyurid populations are reported to increase in response to higher rainfall (Bakker, Patterson, et al. 2024; Bennison, Godfree, and Dickman 2018; Greenville et al. 2016). Therefore, it seems plausible that despite declines in relative abundance as prey, the populations of dasyurids at Toorak may be stable or even have increased across 2023–2024. If so, the increased abundance of rodents simply outweighed the contribution of dasyurids as owl prey during the plague period (Bennison, Godfree, and Dickman 2018; Clulow et al. 2011; Heywood and Pavey 2002; Spencer, Newsome, and Dickman 2017).

The native mouse, *L. forresti* , is known to be distributed patchily throughout its broad geographic range, although it is occasionally locally plentiful (Baker and Gynther 2023; Dickman, Leung, and Van Dyck 2000; Madani 2022). This rodent species may fluctuate in response to rainfall driven resource pulses, but it is unclear if it is irruptive (Madani 2022). *Leggadina forresti* formed a notable component of pellets assessed by Woolley (2010), but their abundance as prey was significantly lower in all collections analysed here. Toorak's habitat may have been locally more suitable, and therefore the species abundant, at the time of Woolley's collections. The introduced house mouse, *M. musculus* , was not found in any present‐day collections, although the species can form the primary component of owl diet in some regions (Bilney 2020; Debus and Tsang 2023; McDowell and Medlin 2009). *Mus musculus* presence may be more closely attributed to human activity (Bilney 2020; McDowell and Medlin 2009), and the relative isolation of Toorak and its closure as a research station (Tapp 2012) may be related to lower present‐day *M. musculus* abundance.

Bird (Class Aves) relative abundance was consistent across all assessed collections other than present‐day Summer, where birds significantly decreased in abundance. This decrease is likely representative of owl prey switching to the more abundant *R. villosissimus* . The most abundant bird species was *Taeniopygia* sp. (finches), which is consistent with the findings of Woolley (2010), who suggested the communal nesting of finches contributes to easier hunting by owls. Both lizards (Order Squamata) and frogs (Order Anura) were rarely found in present‐day collections, despite being found in larger numbers by Woolley (2010). Frogs may be intensively selected as prey during wet periods (Valente 1981), while lizards may at times form a key component of owl diets (Bilney 2020). Nevertheless, these species are generally not the primary prey of eastern barn owls (e.g., Debus, Olsen, and Rose 2004; Schoenefuss et al. 2024), and their abundance in the Historic dataset is likely representative of diet flexibility related to a local temporal abundance, which was not observed during the present‐day collections (Valente 1981; Woolley 2010).

### 4.4 Comparison of Community Diversity

Species richness alone underestimated the true small mammal community structure observed in the present study. Species richness was the same across all present‐day collections (five species), while the Historic collection (Woolley 2010) was higher (six species), representing the addition of *M. musculus*. When considering only species richness, an interpretation that the community structure was consistent across the present‐day collections seems appropriate. However, this is not reflective of notable, and at times significant, differences in small mammal relative abundance observed across the four assessed collections. Integrating Hill numbers into diversity estimates, as outlined by Chao et al. (2014), enabled incorporation of individual counts and therefore offered further insight into community structure. Under Hill‐Shannon diversity, which remains sensitive to rare species (Roswell, Dushoff, and Winfree 2021), collections that encompassed the *R. villosissimus* plague had an expected lowest diversity, as other species became less abundant as prey. Woolley (2010) found a relatively even spread across mammal taxa, and hence, the Historic collection was more diverse than the Initial collection, which was comparatively dominated by *S. douglasi* . Comparable results, albeit with lower estimates, were presented for Hill‐Simpson curves, which are primarily sensitive to dominant species (Roswell, Dushoff, and Winfree 2021). Under this metric, Initial replaced Historic as the most diverse collection, likely due to its dominance of primarily *S. douglasi, R. villosissimus* and to a lesser extent *Planigale* spp. Summer remained the least diverse under this metric, reinforcing the dominance of *R. villosissimus* as prey during a plague.

### 4.5 Implications

Using owl pellets, we have demonstrated a shift in small mammal community structure at Toorak. Rainfall is a known driving factor for many rangeland mammal communities (Morton, Gillam, and Thornley 2022), and both rodents and dasyurids may respond positively to rainfall driven resource pulses, albeit to differing degrees (Bennison, Godfree, and Dickman 2018; D'Souza et al. 2013; Greenville et al. 2016). The observed shift from *S. douglasi* to *R. villosissimus* dominance in owl pellets is likely due to a *R. villosissimus* irruption in response to rainfall experienced within the region across 2023–2024. Continued owl pellet monitoring at Toorak offers an unique opportunity to monitor both *S. douglasi* and the broader small mammal community as the rodent plague subsides, while additionally building a long‐term dataset spanning multiple seasons and potential El Niño/La Niña oscillations.

Owl pellet analysis has been widely applied for monitoring temporal changes in small mammal communities (e.g., Bilney, Cooke, and White 2010; Stefke and Landler 2020; van Strien et al. 2015), and the present study further endorses its utility. If roost sites can be located, owl pellets may provide a highly efficient and effective technique for monitoring not just *S. douglasi* but other small mammal species, particularly when environmental conditions may limit the effectiveness of traditional methodologies.

## Author Contributions

**Cameron L. Charley:** conceptualization (equal), data curation (lead), formal analysis (lead), methodology (equal), writing – original draft (lead), writing – review and editing (equal). **Emma L. Gray:** conceptualization (equal), data curation (supporting), methodology (equal), project administration (equal), supervision (equal), writing – review and editing (equal). **Andrew M. Baker:** conceptualization (equal), data curation (supporting), funding acquisition (lead), methodology (equal), project administration (equal), supervision (equal), writing – review and editing (equal).

## Conflicts of Interest

The authors declare no conflicts of interest.

## Data Availability

The authors confirm that the data supporting the findings of this study are available within the article [and/or] its Supporting Information. Raw data and the related code are available via the Zenodo open access repository: https://doi.org/10.5281/zenodo.14625085.

## Appendix 1 Identification Features of Small Mammals Collected From Owl Pellet Material

Terminology followed Archer (1981), other than premolar numbering (Archer's P4 is designated as P3, here). Tooth size and row length were primarily used to distinguish between *Sminthopsis douglasi* (Figure 2A.i,ii), *Sminthopsis macroura* (Figure 2A.iii) and *Sminthopsis crassicaudata* (Figure 2A.iv). Tooth size was markedly larger (longer and wider) in *S. douglasi* than all other examined *Sminthopsis*. Archer (1981) described the dentary length of *S. douglasi* as between 22 and 23.5 mm (albeit based on only a few specimens), with no overlap between *S. macroura* (< 20.2 mm) and *S. crassicaudata* (< 19.7 mm). These lengths were used to corroborate identification. Often owl pellet dasyurid specimens were structurally damaged and missing the incisor teeth. However, in most cases, mandibles that had > 20 mm dentary lengths could be readily attributed to *S. douglasi* , considering the addition of the missing portions contribute upwards of ~2 mm of length and other matching dental features (mandible size, entoconid condition, etc.). *S. douglasi* dentary lengths were also frequently found to be larger than those observed by Archer (1981), often > 24 mm and in some cases ~28 mm (see Figure 2A.ii).

For all *Sminthopsis* (dunnarts), incomplete emergence of premolar tooth 3 (P_3_) (where P3 ≤ P2 in crown height) were denoted as juvenile (Bakker, Patterson, et al. 2024). Because of their smaller mandibles, juvenile *Sminthopsis douglasi* (Figure 2A.i) may be initially confused with adult *Sminthopsis macroura* (Figure 2A.iii) and *Sminthopsis crassicaudata* (Figure 2A.iv), although the tooth row of the juvenile *S. douglasi* (Figure 2E.i) matches the adult (Figure 2E.ii), albeit with a minimal gap between molar 4 (M_4_) and the anterior border of ascending ramus. In juveniles, often M_4_ is inflected in relation to the rest of the dental row. Juvenile *S. douglasi* tooth sizes are comparable to adult specimens, as post‐emergence, mammal teeth do not grow (Luckett and Woolley 1996).

Hypocristid‐entoconid condition was used to distinguish between *Sminthopsis macroura* (Figure 2F.i,ii) and *Sminthopsis crassicaudata* (Figure 2F.iii) based on Archer (1981) (Figure 2G). In *S. crassicaudata* , the hypocristid clearly contacts the entoconid. In *S. macroura* , the hypocristid is clearly separated from the entoconid, approaching the hypoconulid. The hypoconulid was generally pronounced in *S. macroura* and diminished in *S. crassicaudata*.

Length and width of molar teeth was used to distinguish between *Rattus villosissimus* (Figure 2B.i,ii), and *Rattus rattus* (Figure 2B.iii), where *R. villosissimus* molars were conspicuously wider and more robust. Also, incisor teeth of *R. rattus* were often thinner than *R. villosissimus* , although this feature alone was not consistent enough to distinguish between species. M_3_ was partly or un‐emerged in juvenile *R. villosissimus* (Musser 2014) (Figure 2C.i) compared to fully emerged as seen in adults (Figure 2C.ii,iii). Tooth wear as an age characteristic (Lidicker 1966) during rodent plagues is not useful, as even large adult rodents typically presented unworn teeth at the time of predation.

To distinguish between smaller rodent species (Figure 2d), size and shape of the teeth, and overall size of the jaw, was used. *Pseudomys desertor* (Figure 2D.i) were larger than all other smaller rodents examined, while *Pseudomys delicatulus* (Figure 2D.v) were smaller than *Leggadina*. *Leggadina lakedownensis* (Figure 2D.ii) and *Leggadina forresti* (Figure 2D.iii,iv) were similar but readily distinguishable based on M_1_ antero‐lingual and antero‐buccal cuspids (Cramb, Price, and Hocknull 2018), which combined form a more squared ‘teardrop’ shape in *L. forresti* .

**TABLE A1:** Vertebrate craniodental material identified from fragmented/loose owl pellet material.

Collection	Species	Type		Count
Initial 2023	*Sminthopsis douglasi* (Adult)	Mandible	Left	15
			Right	17
		Top jaw	Left	19
			Right	21
		Skull	Complete	14
			Fragment	14
	*Sminthopsis douglasi* (Juvenile)	Mandible	Left	4
			Right	5
	*Sminthopsis macroura* (Juvenile)	Mandible	Left	1
	*Planigale* spp.	Mandible	Left	1
			Right	1
		Top jaw	Left	1
			Right	1
	*Rattus villosissimus* (Adult)	Mandible	Left	7
			Right	9
		Skull	Complete	6
			Fragment	3
	Family Agamidae	Mandible		3
	*Taeniopygia* sp.	Beak	Lower	6
			Upper + skull	3
	*Melopsittacus undulatus*	Skull	Complete	3
		Beak	Lower	3
			Upper	3
	*Mirafra javanica*	Skull	Complete	1
		Beak	Lower	3
			Upper	1
	*Artamus* sp.	Skull	Complete	1
		Beak	Lower	2
	Class Aves	Skull		1
Winter 2023	*Sminthopsis douglasi* (Adult)	Mandible	Left	2
			Right	2
	*Sminthopsis macroura* (Juvenile)	Mandible	Left	3
			Right	3
	*Sminthopsis* sp.	Top Jaw	Left	1
			Right	1
	*Rattus villosissimus* (Adult)	Mandible	Left	5
			Right	5
	*Rattus villosissimus* (Juvenile)	Mandible	Left	1
	*Planigale* spp.	Mandible	Left	5
			Right	4
	Class Aves			3
	*Melopsittacus undulatus*	Beak	Lower	2
	Order Anura	Ilia		1
Summer 2024	*Sminthopsis douglasi* (Adult)	Mandible	Left	1
			Right	1
	*Rattus villosissimus* (Adult)	Mandible	Left	1
			Right	2
